# Regulation of idiopathic pulmonary fibrosis: a cross-talk between TGF-*β* signaling and MicroRNAs

**DOI:** 10.3389/fmed.2024.1415278

**Published:** 2024-09-25

**Authors:** Shuo Wang, Hanming Yu, Shi Liu, Yuqing Liu, Xiu Gu

**Affiliations:** Department of Pulmonary and Critical Care Medicine, The Fourth Affiliated Hospital of China Medical University, Shenyang, China

**Keywords:** transforming growth factor-*β*, MicroRNAs, pulmonary fibrosis, targeted therapy, feedback loop

## Abstract

Pulmonary fibrosis (PF) is a highly complex and challenging disease affecting the respiratory system. Patients with PF usually have an abbreviated survival period and a consequential high mortality rate after the diagnosis is confirmed, posing serious threats to human health. In clinical practice, PF is typically treated by antifibrotic agents, such as Pirfenidone and Nintedanib. However, these agents have been reported to correlate with substantial adverse effects, escalating costs, and insufficient efficacy. Moreover, it remains unclarified about the multifactorial pathology of PF. Therefore, there is an urgent demand for elucidating these underlying mechanisms and identifying safe, efficient, and targeted therapeutic strategies for PF treatment. The crucial role of the transforming growth factor-*β* (TGF-β) signaling pathway in PF development has been explored in many studies. MicroRNAs (miRNAs), which function as post-transcriptional regulators of gene expression, can significantly affect the development of PF by modulating TGF-*β* signaling. In turn, TGF-β signaling can regulate the expression and biogenesis of miRNAs, thereby substantially affecting the progression of PF. Hence, the therapeutic strategies that focus on the drug-targeted regulation of miRNAs, either by augmenting down-regulated miRNAs or inhibiting overexpressed miRNAs, may hinder the pathways related to TGF-*β* signaling. These strategies may contribute to the prevention and suppression of PF progression and may provide novel insights into the treatment of this disease.

## 1 Introduction

Pulmonary fibrosis (PF) encapsulates a range of persistent and progressively deteriorating lung disorders that incrementally affects the interstitium. This disease may induce hampered gas exchange, breathlessness, and compromised quality of life, ultimately resulting in respiratory failure and death (1). As the most prevalent subtype of PF, idiopathic pulmonary fibrosis (IPF) is an chronic, progressive, and fibrotic disorder in the interstitial lung (2). Globally, the estimated incidence of IPF ranges from 0.09 and 1.30 per 10,000 individuals, while the average age of patients with IPF is approximately 65–70 years, presenting a significantly higher incidence with an increase in age (3). In the context of the aging population worldwide, the burden of PF on patients and healthcare systems is predicted to rise progressively. According to current treatment guidelines, Pirfenidone and Nintedanib are recommended in the treatment of PF (4). However, the efficacy of these agents in decelerating disease progression and enhancing the quality of life is limited to a certain extent. Additionally, they are often associated with gastrointestinal tolerability concerns (5–7). Therefore, the development of novel drug therapies is of paramount importance for the treatment of this disease.

IPF is characterized by the excessive production and disorganized deposition of extracellular matrix (ECM) components, resulting in irreversible structural distortions and loss of organ function (8). It is now widely recognized that IPF arises from a complex interplay of genetic and environmental risk factors, with repetitive localized microinjuries to the senescent alveolar epithelium playing a central role. These micro-injuries initiate aberrant epithelial–fibroblast communication, the induction of matrix-producing myofibroblasts, and considerable extracellular matrix accumulation and remodelling of lung interstitium (2).

Transforming growth factor-*β* (TGF-β) is a pivotal mediator in fibrogenesis (9, 10). There are three TGF-β isoforms (TGF-β1, β2, and β3), alongside three cell-surface receptors - Type I (TGFBR1), Type II (TGFBRI2), and Type III (TGFBR3), all of which contribute to the TGF-*β* signaling pathway (11). Triggered by the ligand-receptor interaction (9), TGF-*β* signaling may be initiated when TGF-β first binds to its specific receptor, TGFBR2, thereby recruiting TGFBR1 into the complex via the unique interface generated by the TGF-*β*-TGFBR2 complex. This forms a heterotetramer complex consisting of a single dimeric TGF-β molecule binding to two TGFBR1 molecules and two TGFBR2 molecules. The formation of this complex leads to the phosphorylation of TGFBR1 by TGFBR2, which subsequently phosphorylates Smad proteins to propagate signals to the nucleus, bind to consensus sequences, and regulate gene transcription (11). As a co-receptor, TGFBR3 promotes the binding of ligands to TGFBR2, which augments TGF-*β* signaling (12). As the downstream components of SMAD signaling, Smad2/Smad3 are hypothesized to be paramount intermediators of TGF-*β* signaling in tissue fibrogenesis. Smad4 can work synergistically with Smad2/3 to facilitate this process, while Smad6 and Smad7 can function as negative regulators to mitigate TGF-*β*-mediated fibrosis (13). Apart from initializing Smad-dependent signaling chains, TGF-β can instigate Smad-independent signaling cascades, including the MAPK family (*Erk* (14, 15), *JNK* (16, 17), and *p38 MAPK* (18, 19)), *Rho-like GTPases* (20, 21), and the *AKT*/phosphatidylinositol-3 kinase pathway (22, 23). IPF is a disease primarily associated with aging, and elevated TGF-*β*1 is a major factor in its pathogenesis (24). TGF-β is released following epithelial cell injury and serves as a central pro-fibrotic growth factor driving the progression of pulmonary fibrosis. Its multifunctional roles include stimulating the proliferation and differentiation of epithelial cells and fibroblasts, activating ECM production in myofibroblasts, catalyzing epithelial-mesenchymal transition, accelerating epithelial apoptosis and cell migration, and inducing the production of connective tissue growth factor (CTGF) along with other mediators such as Fibroblast Growth Factor (FGF), Insulin-like Growth Factor (IGF), and Platelet-Derived Growth Factor (PDGF) (25).The heightened level of TGF-β1 has been observed in both animal models of IPF and tissue samples from IPF patients (26). The overexpression of active TGF-*β*1 may result in the progression of PF (27), whereas inhibiting TGF-β signaling can mitigate PF in animal models (28).

MicroRNAs (miRNAs) are a highly conserved group of small single-stranded non-coding RNAs measuring between 19 to 25 nucleotides. They can modulate genes at both the transcriptional and post-transcriptional levels by binding to the 3′-untranslated region (UTR) of target mRNAs (29, 30). In animals, miRNA gene transcription, facilitated by RNA polymerase II (pol II), first yields extended primary transcripts (pri-miRNAs). These pri-miRNAs are subsequently cropped by the RNase-III enzyme Drosha to produce hairpin intermediates (pre-miRNAs) located within the nucleus. Drosha can be fused with its indispensable cofactor DGCR8/Pasha into a large ensemble (500-650 kDa) named the microprocessor complex (31). DGCR8/Pasha, accounting for approximately 120 kDa in size, is equipped with two dsRNA-binding structural domains. Thereafter, the pre-miRNA is shuttled into the cytoplasm through exportin-5, a constituent of the Ran-dependent nuclear transporter receptor family. Upon reaching the cytoplasm, pre-miRNAs are finally processed into approximately 22-nucleotide miRNA duplexes by the cytoplasmic RNase-III protein Dicer, but their cellular persistence is typically transient. Typically, one strand of this transient duplex would degrade while the other would transform into the mature miRNA. The strand choice for retention is determined by the relative thermodynamic stability of both ends of the duplex (32). Dicer, a cog in the miRNA machinery, is known to form interactions with a variety of proteins, each imparting different functions in miRNA stability, effector complex formation, and operational actions. For instance, human AGO2, a member of the Argonaute protein family, has been recently validated to function as a “slicer” enzyme, which can cleave target mRNAs (33, 34).

Mature miRNAs are sorted into exosomes or microvesicles (35), and extracellular miRNAs can also be loaded into high-density lipoproteins (HDL) (36, 37) or bound to AGO2 proteins outside of vesicles (38). These three mechanisms protect miRNAs from degradation and ensure their stability. Given the transporter capability of vesicles, the role of miRNAs in exosomes has garnered increasing attention. Since exosomal miRNAs can stably persist in the blood, urine, and other body fluids of patients, exosomes can reflect their tissue or cellular origin through the presence of specific surface proteins. The use of exosomes and their cargo miRNAs as clinical tools for diagnosing and monitoring diseases holds substantial promise and may even be beneficial for gene therapy in certain conditions.It has been shown that HDL can deliver miRNAs to recipient cells and alter gene expression. For example, HDL delivery of miR-223 significantly reduced EFNA1 and RhoB mRNA levels in hepatocytes (36). Manipulation of HDL-miRNA levels and the use of HDL as a delivery vehicle for RNA and chemicals have demonstrated potential in the treatment of cardiovascular disease. HDL-miR-223 mimic approaches can be used to prevent or treat hypercholesterolemia (hepatic cholesterol biosynthesis) and endothelial activation (monocyte and neutrophil adhesion) and attenuate atherosclerosis (39).

It has been recently revealed that there is a strong association between numerous miRNAs and tissue or organ fibrogenesis (40–43). Promising results have been elicited from miRNA mimics and miRNA inhibitors, and they are expected to become novel therapeutic agents, which are currently under preclinical development (44). KADOTA et al. corroborated the antifibrotic properties of miR-16, miR-26a, miR-26b, miR-141, miR-148a, and miR-200a located in human bronchial epithelial cell-derived extracellular vesicles (HBEC EVs). They further proposed that the introduction of these HBEC EVs may hold promise as an antifibrotic strategy in the treatment of IPF through miRNA-mediated curbs on TGF-*β* signaling (45).

## 2 The interaction between TGF-β signaling and MiRNAs

TGF-β signaling can exert impacts on the expression of miRNAs while concurrently being regulated by these miRNAs. Moreover, a majority of the classical TGF-*β* signaling pathway constituents may be susceptible to miRNA impacts. Specifically, miR-424 enhances profibrotic properties via stimulating the TGF-*β*/Smad pathway, which can be attributed to the direct engagement with TGIF2, an intrinsic repressor of TGF-β signaling. This further results in sub-mucosal fibrosis within the oral mucosa (46). On the contrary, miR-130a also presents antifibrotic properties by modulating the expression of TGFBR1 via directly targeting TGFBR1, thus inhibiting cardiac fibrosis after myocardial infarction (47). Additionally, miR-326 is validated to correlate with TGF-β1 3’UTR, which inhibits the expression of TGF-*β*1 at the post-transcriptional stage, thereby mitigating PF (48).

Furthermore, the molecules involved in TGF-β signaling are capable of modulating the expression and biogenesis of miRNAs. The biogenesis of miRNAs encompasses synchronized processes, including miRNA transcription and the consequent processing and maturation of miRNA units. Davis et al. uncovered that TGF-*β* signaling enhanced the processing of the primary transcript of miR-21 (pri-miR-21) into the precursor miR-21 (pre-miR-21) through the DROSHA complex (49). Receptor-activated Smad (R-Smad) proteins that are exclusive to TGF-*β* signaling are enlisted to the RNA deconjugating enzyme *p68* complex (also termed *DDX5*) of the ‘pri-miR-21’, where they facilitate the maturation of miRNAs by managing DROSHA, which is a constituent of the DROSHA microprocessor complex.

Marquez et al. reported a unique positive feedback loop, in which TGF-*β* stimulated the processing of the primary miR-21 precursor (pri-miRNA) into mature miR-21 (50). Interestingly, the mature miR-21 suppressed TGF-*β* signaling, but the R-Smad protein was also conscripted to the primiR-21 RNA deconjugating enzyme p68 complex. The suppression of SMAD7, a TGF-β signaling inhibitor, can be induced by mature miR-21, thereby facilitating fibrosis. It has been confirmed in numerous studies that the expression of various miRNAs can be altered in different cell types after the treatment with TGF-*β* family ligands. For example, Ottaviani et al. found that TGF-β incited the lncRNA MIR100HG through SMAD2/3 transcription, which housed miR-100, miR-125b, and let-7a within its intron (51). Additionally, Yang et al. observed that miR1306 negatively influenced TGF-*β*/Smad3 signaling by targeting *TGFBR2* (52). In contrast, SMAD4 was directly associated with the miR-1306 promoter, thereby inhibiting its transcriptional activity.

**Figure 1:**
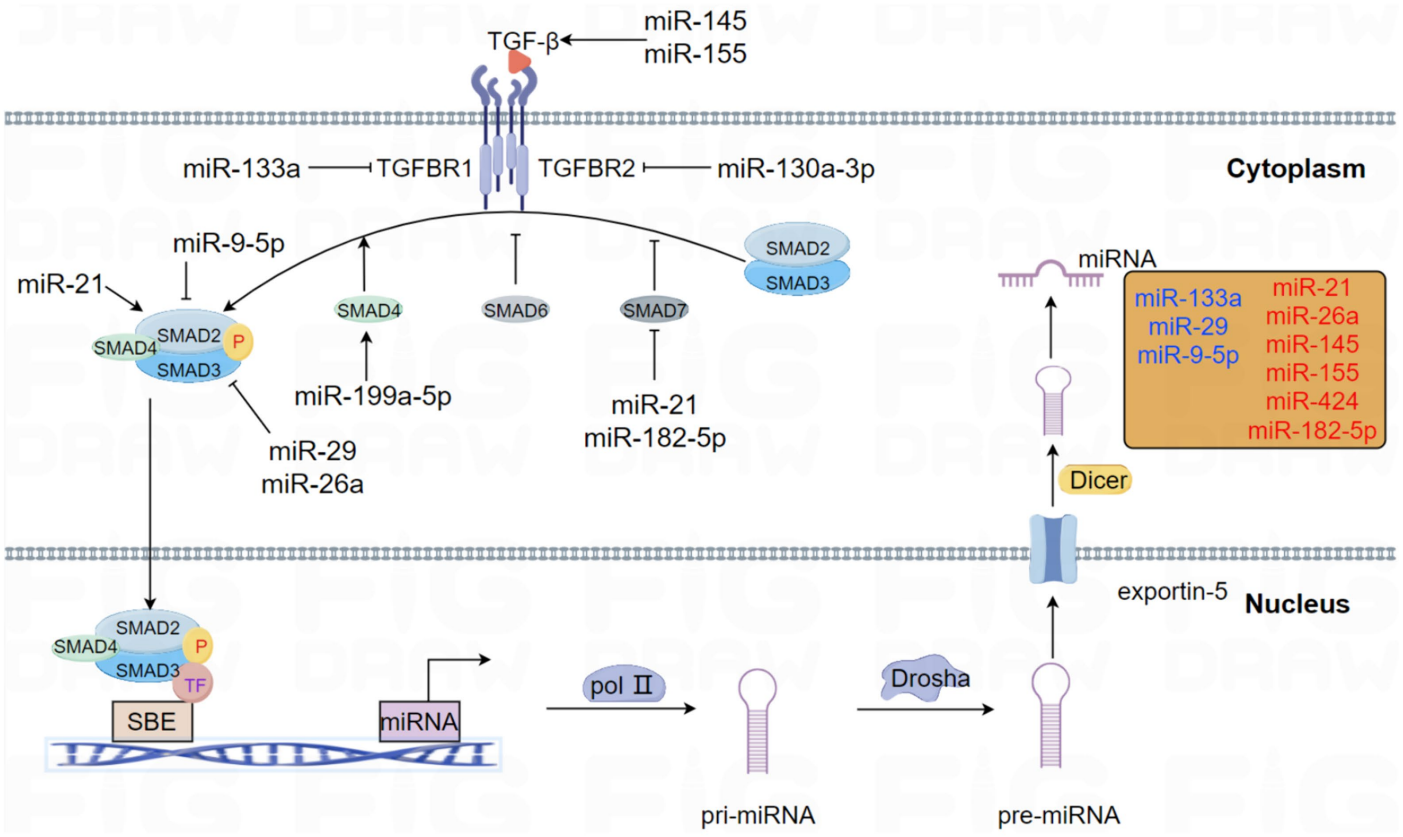
This schema elucidates the role of miRNAs that modulate the pivotal molecules involved in the TGF-β signaling pathway in PF. Likewise, the central molecules of the TGF-β signaling pathway have interactive roles in regulating miRNA expression and biogenesis. Emphasizing the presence of feedback loops, miRNAs involved in forming negative and positive feedback with the TGF-β signaling pathway are specifically highlighted in different colors. Specifically, blue represents negative feedback loops, whereas red represents positive feedback pathways.

The connection between TGF-*β* signaling and miRNA mechanisms has been proved in many studies. In this review, the acting patterns of this interaction and their functions in the occurrence and progression of PF are summarized. This interaction is illustrated in the following Figure 1.

## 3 MiRNAs impeding the progression of PF

### 3.1 MiR-133a

MiR-133a is mainly expressed in muscle tissues (53) predominantly conserved in skeletal and cardiac muscles. It plays a critical role in various physiological processes, such as fibrosis (54, 55), myocardial infarction (56), scarring (57), cancer (58), and bone metabolism (59). It has been recently revealed that the expression of miR-133a is considerably down-regulated in the lungs of patients with IPF compared with age-matched males without fibrotic lung diseases (60). Wei et al. unveiled a novel role of miR-133a as a negative feedback regulator in the profibrotic signaling pathway of TGF-β1 (55). MiR-133a can impede the differentiation of myofibroblasts by targeting multiple constituents of the profibrotic pathway of TGF-β1, including TGFBR1, CTGF, and collagen type 1-alpha1 (Col1a1). Besides, TGF-β1 promotes the transcription of miR-133a through the activation of both conventional (Smad3) and unconventional (p38MAPK) signaling pathways. This instigates a negative feedback loop, thereby inhibiting the progression of PF. Intriguingly, the overexpression of miR-133a has been found to alleviate PF induced by bleomycin in mice, underscoring its potential therapeutic utility (55) (Table 1).

**Table 1:** Impact of miRNAs in pulmonary fibrosis.

MicroRNA	Brief role in fibrosis	Molecular target	Organism	Reference
miR-133a	Protective	TGFBR1, CTGF, Col1a1	Human, mouse	(55)
miR-29	Protective	TGF-β, CTGF, Smad3	Mouse	(63)
miR-130a-3p	Protective	TGFBR2	Mouse	(71, 72)
miR-200	Protective	ZEB1, ZEB2/SIP1	Human	(75)
miR-9-5p	Protective	TGFBR2, p-Smad2, NOX4	Human	(79, 80)
miR-26a	Protective	p-samd3	Human, mouse	(84)
miR-21	Enhancing	Smad7, p-Smad2	Mouse	(95)
miR-145	Enhancing	α-SMA	Mouse	(100)
miR-155	Enhancing	TGF-β1	Human	(102)
miR-424	Enhancing	Slit2, Smurf2	Human	(110, 111)
miR-182-5p	Enhancing	Smad7	Human, mouse	(112)
miR-199a-5p	Enhancing	CAV1, Smad4	Human, mouse	(117)

### 3.2 MiR-29

The miRNA-29 family, including miR-29a, miR-29b, and miR-29c, has been confirmed to have a pivotal role in the fibrosis process within several organs, thus, garnering interest as a potential antifibrotic regulator (61). CUSHING et al. reported a negative correlation of the level of miR-29 with the expression of profibrotic genes and the severity of fibrosis. In IMR-90 cells, a fetal lung fibroblast cell line commonly utilized in fibrosis molecular mechanism studies, miR-29 is suppressed by TGF-*β*, and many fibrosis-related genes normally up-regulated by TGF-β are derepressed after the knockdown of miR-29. Remarkably, it has been demonstrated in a comparative analysis between TGF-β and miR-29 targets that miR-29 can independently regulate different subsets of profibrotic genes, such as laminin, integrins, *ADAMTS9, ADAM12, NID1 gene*, and integrin ITGA11 (62). Xiao et al. found that miR-29 was negatively regulated by TGF-*β*/Smad signaling during fibrosis, with Smad3 identified as a downstream target. Notably, the overexpression of miR-29 could inversely regulate the expression of TGF-β, connective tissue growth factors (CTGFs), and Smad3 signaling. Importantly, non-invasive miR-29 gene therapies based on the SB transposon system have been developed for the treatment of PF. These therapies could enhance the expression of miR-29 in normal mouse lungs to prevent bleomycin-induced fibrosis. Besides, they can also significantly restore the higher level of miR-29 in fibrotic lungs, thus avoiding the progression of PF in mouse models (63). Chioccioli et al. uncovered that the decreased level of miR-29 in the peripheral blood of IPF patients was correlated with the poor prognosis and significantly decreased survival rate of these patients (64). Astonishingly, the supplementation with the miR-29 mimic, MRG-229, can mitigate the up-regulation of fibrosis-associated genes instigated by TGF-*β* and inhibit the synthesis and secretion of collagen in both standard and IPF cells *in vitro*. Moreover, similar outcomes are also observed in bleomycin-induced PF rats and non-human primate models, indicating an improvement in fibrosis. These findings may support the exploration of miR-29 as an innovative and potentially effective therapy for lung diseases characterized by fibrosis.

Interestingly, IPF is characterized by the progression from the periphery toward the center. This disease presents distinct pathological characteristics in different lung regions that may resemble varying stages of the disease, hence it can be considered a heterogeneous and progressive disease (65).

McDonough et al. demonstrated that the expression of many collagen genes in the lungs of IPF patients, least affected by the disease, was up-regulated significantly before observable changes in protein levels. Simultaneously, a decrease in the miRNA level of miR-29 was also observed at this stage, implying that IPF patients, even with normal histology, may exhibit molecular abnormalities (66). These findings may potentially assist in implementing more effective diagnostic measures or tailoring more efficient therapies for patients with PF.

### 3.3 MiR-130a-3p

The close association between miR-130a-3p and fibrosis across various tissues has been reported in many studies. Specifically, miR-130a-3p can bind to MAPK1, TGFBR1, and TGFBR2 and inhibit their expression, a mechanism that contributes to alleviating hepatic fibrosis (67). However, in the context of obstructive nephropathy, miR-130a-3p directly targets the 3’-UTR of *SnoN* to suppress the expression of *SnoN*, thereby inadvertently promoting the activation of TGF-β1/Smad signaling related to renal fibrosis (68). The correlation of miR-130a-3p with lung inflammation (69) and airway remodeling (70) has been demonstrated in some studies. In the previous, TGFBR2 was identified as a potential target of miR-130a-3p. Besides, the interaction of miR-130a-3p/TGFBR2 axis can inhibit the differentiation of lung fibroblasts via the TGF-β1/Smad signaling pathway. Additionally, it has been found that the down-regulated expression of miR-130a-3p can affect the secretion of inflammatory cytokines (including IL-1β, IL-6, TNF-*α*, and TGF-β1) in the inflammatory and fibrosis phases and the deposition of the extracellular matrix (α-SMA, FN, HYP, and collagen). Overall, these findings suggest that miR-130a-3p can exert anti-inflammatory and antifibrotic effects in the treatment of PF (71, 72).

### 3.4 MiR-200

Among vertebrates, the miR-200 family is deemed one of the most conserved miRNAs, encompassing five members: miR-200a, miR-200b, miR-200c, miR-141, and miR-429. This family holds a central role in establishing epithelial phenotypes during embryogenesis and organogenesis (64). Yang unraveled the significant down-regulated expression of miR-200a, miR-200b, and miR-200c in the lungs of mice affected by experimental PF. Concurrently, the expression of miR-200a and miR-200c was down-regulated in the lungs of IPF patients. Interestingly, it was validated that the members of the miR-200 family were inversely correlated with the TGF-*β*1-induced EMT, and miR-200c even demonstrated the ability to attenuate PF in mice (73). As revealed in recent studies, miR-200a exhibits an antifibrotic effect by inhibiting the profibrotic TGF-*β*/SMAD-3/*α*-SMA pathway (74). Moreover, the expression of ZEB1 is up-regulated by the TGF-β pathway in IPF. Hence, it can be speculated that miR-200 s can potentially govern the EMT in IPF by targeting and suppressing *ZEB1* and *ZEB2/SIP1* (75). These findings could pave the way for future therapeutic strategies targeting miR-200 s in the treatment of IPF.

### 3.5 MiR-9-5p

The dysregulation of miR-9-5p has been found to correlate with fibrosis in various tissues and organs (76–78). It has been corroborated that the expression of miR-9-5p is down-regulated in PF tissues compared with normal physiological conditions. Interestingly, the overexpression of miR-9-5p has been found to suppress the expression of TGF-β1 and p-Smad2 (79). Recently, Fierro et al. identified a feedback loop between TGF-*β*1 and miR-9-5p, demonstrating an intriguing interaction. They found that TGF-β1 up-regulated the expression of miR-9-5p through a mechanism dependent on reactive oxygen species (ROS), while miR-9-5p was found to negatively regulate the TGF-β signaling pathway by targeting TGFBR2 and NADPH oxidase 4 (NOX4). These data prove that miR-9-5p exerts antifibrotic effects in a bleomycin-induced PF animal model. Notably, miR-9-5p delays the TGF-β1-dependent transformation of lung fibroblasts into myofibroblasts by inhibiting the phosphorylation of Smad2 and the nuclear translocation of Smad2/3 (80). These findings highlight the complex and dynamic interplay of miR-9-5p in the modulation of fibrosis, offering an intriguing target for the therapeutic intervention of PF.

### 3.6 MiR-26a

MiR-26a has been reported to play an important role in the regulation of many diseases, such as renal tubulointerstitial fibrosis, lens fibrosis, and cataract (81, 82). MiR-26a can regulate the expression of let-7d by targeting Lin28B, which contributes to the regulatory effect of miR-26a on EMT, and ultimately, may enhance the anti IPF This could help miR-26a to regulate EMT and ultimately enhance the anti-fibrotic activity of miR-26a against IPF (83). LIANG found that overexpression of miR-26a inhibited TGF-β1-induced fibrosis in MRC-5 cells and attenuated experimental pulmonary fibrosis in mice. More importantly, the positive feedback loop between miR-26a and p-Smad3 was associated with pulmonary fibrosis. TGF-β1 negatively regulated miR-26a expression by regulating Smad3, and miR-26a inhibited the nuclear translocation of p-Smad3 in order to block signaling events downstream of TGF-β1, which ultimately attenuated collagen deposition and mitigated pulmonary fibrosis (84).

## 4 MiRNAs facilitating the progression of PF

### 4.1 MiR-21

MiR-21 has been implicated in an array of lung diseases, including acute lung injury/acute respiratory distress syndrome (85), asthma (86), PF (87), and lung cancer (88). An increase in the expression level of miR-21 has been documented in both the PF mouse model (in the bleomycin model) and tissue samples in IPF patients (89, 90). Similarly, Zhou et al. observed that TGF-β1 induced the expression of miR-21 upon the interaction with TGFBR1 through the binding of SMAD3 or SMAD2/3/4 complexes to the miR-21 promoter region. This process culminates with the promotion of ECM protein deposition in both human lung fibroblasts IMR-90 and a mouse model related to bleomycin-induced PF. This implies a novel molecular mechanism working via the positive feedback axis of TGFβ1/SMADs/miR-21 (91).

Sato et al. analyzed the influence of TGF-β1 and tissue stiffness on the expression of miR-21 in rat lung fibrocytes and human circulating fibrocytes. They found that fibrocytes treated with varying concentrations of TGF-β1 and cultured on plates exhibited different levels of stiffness. Fibrocytes were cultivated on soft (1 kPa) plates to simulate the characteristics of normal pulmonary tissue as well as on stiff (50 kPa) plates to replicate the conditions in fibrotic lung tissues. Interestingly, the substrate stiffness changes miR-21 expression in fibrocytes. Culturing lung fibrocytes on hard plates increased miR-21 expression compared to culturing lung fibrocytes on soft plates (92).

Radiation-induced lung fibrosis (RILF) is one of the most severe side effects of lung cancer radiotherapy on normal tissues. It poses a considerable challenge to the broader application of radiotherapy in treating lung cancer patients (93, 94). It has been revealed that the expression of miR-21 is significantly up-regulated in regions affected by radiation injury. Concomitantly, miR-21 has been found to down-regulate the expression of Smad7 while amplifying the phosphorylation of Smad2. This, in turn, propels the activation of Smad-dependent TGF-*β* signaling, a noteworthy contributor to the onset of RILF. Hence, the targeted mitigation of miR-21 in the affected regions may hold potential as an effective therapeutic strategy in preventing the progression of RILF (95).

### 4.2 MiR-145

MiR-145 is highly expressed in blastomeres, which constitute a collection of pluripotent stem cells. The primary function of miR-145 is to inhibit the self-renewal of embryonic stem cells (ESCs), thereby inducing lineage-specific differentiation. Specifically, miR-145 has been observed to reprogram fibroblasts into smooth muscle cells during development (96–98). As reported in recent studies, patients with COVID-19 and IPF may exhibit the mutual up-regulation of miRNAs, including miR-145-5p, which may play a role in the progression of PF (99). Yang et al. identified that the level of the primary transcript of the miR-145 cluster gene (pri-miR-145) increased in TGF-β1-treated lung fibroblasts. This implied that the up-regulated expression of mature miR-145 may be attributed to the enhanced transcription of the miR-145 gene cluster. Besides, they also confirmed that miR-145 played a pivotal role in promoting the progression of PF by reinforcing the activation of latent TGF-β1. MiR-145 can fulfill this function by up-regulating the expression of *α*-SMA, thereby boosting the contractility of myofibroblasts. Intriguingly, the mechanical forces presented by cell contraction can act as a trigger to activate latent TGF-β1. It has further been observed that the knockout of miR-145 protected mice from bleomycin-induced PF, suggesting that miR-145 could be targeted as a potential treatment strategy for resisting pathological fibrotic diseases (100).

### 4.3 MiR-155

As a principal miRNA, miR-155 has been validated to play a pivotal role in the development of fibrosis, with persistent up-regulated expression observed across various fibrotic diseases (101). Notably, miR-155 has been confirmed to enhance collagen synthesis in animal models related to fibrosis and in human fibroblast cell lines derived from fibrotic tissues, thus exacerbating fibrosis. Further, it has been identified that miR-155 is instrumental in the activation of TGF-*β*1 in fibroblasts and macrophages, offering a potential alternative target for the treatment of fibrotic lesions (102). Kong et al. demonstrated that the TGF-β/Smad4 pathway incited the activity and expression of the miR-155 promoter. This, in turn, stimulates the TGF-β-induced EMT and the dissolution of tight junctions, as well as encouraging cell migration and invasion (103). Sun et al. unraveled that using a miR-155 antagonist could significantly mitigate histological changes and decrease hydroxyproline levels in a mouse model related to bleomycin-induced PF. The inhibition of miR-155 can down-regulate the expression of IL-4, TGF-*β*, and interferon-*γ* after BLM treatment. It was further observed that the mitogen-activated protein kinase signaling pathway of TGF-β-activated kinase 1/MAPK 7 (MAP3K7)-binding protein 2 (TAB2) was activated by BLM. However, this process could be suppressed by the miR-155 antagonist. The *in vivo* application of the antagomir-155 can mitigate BLM-induced pathological changes and may, therefore, represent a promising therapeutic strategy for PF (104).

Due to the central role of miRNAs in the immune system, miR-155-5p is intimately involved in the inflammatory response. Capsaicin (Cap) quenched the inflammatory response in a rat model of bleomycin-induced PF by interrupting the IL-1β, TNF-*α*, and TGF-β1 pathways through down-regulating the expression of miR-155-5p. Hence, IPF-related lung injury may be ameliorated by regulating the expression of miR-155 (105).

### 4.4 MiR-424

MiR-424-5p has been validated to correlate with the occurrence, progression, treatment, and prognosis of tumors, embodying either the role of an oncogene or a tumor suppressor gene (106–108). The results of recent studies have provided novel insights into the function of miR-424 in the landscape of PF. The elevated level of miR-424 has been detected in the extracellular vesicles of lung fibroblasts originating from IPF patients (109). Moreover, a 1.7-fold increase in miR-424 expression was also observed in human fibrotic lung tissues, and a notable 2.6-fold increase was documented in TGF-*β*1-treated HLFs, compared with non-fibrotic lung tissues. MiR-424 can exert profibrotic effects by targeting the expression of Slit2, a prohibitive regulator that can curb profibrotic signaling of TGF-*β*1 and function as an inhibitor of the TGF-β1 signaling pathway (110). Xiao revealed that TGF-*β* propagated the expression of miR-424, which in turn suppressed Smurf2, a negative regulator of TGF-*β* signaling. Intriguingly, the silencing of Smurf2 by miR-424 augmented the activity of Smad3, thereby cementing the significance of miR-424 in TGF-β signaling. Overall, miR-424 orchestrates a positive feedback loop in the TGF-β signaling pathway, which promotes the differentiation of myofibroblasts in IPF (111).

### 4.5 MiR-182-5p

MiR-182-5p is markedly over-expressed in the lung tissues of mice suffering from BLM-induced fibrosis. TGF-β1 is a protein with known profibrotic effects, and it can induce the over-expression of miR-182-5p. Interestingly, miR-182-5p plays a role in the down-regulation of Smad7, which is specifically targeted by miR-182-5p, and this negative regulation results in the exacerbation of PF (112). As a major checkpoint in the TGF-β1/Smad signaling pathway, Smad7 is a negative regulator that can inhibit the phosphorylation of Smad2/Smad3, thereby interrupting TGF-β1 signaling (113). More importantly, miR-182-5p forms a positive feedback loop, a mechanism that expedites the deterioration of PF. By diminishing the level of miR-182-5p, it was observed that PF was alleviated through the suppression of profibrotic proteins (namely, fibronectin, *α*-smooth muscle actin, and p-Smad2/p-Smad3) and the potentiation of Smad7. Remarkably, the concurrent inhibition of miR-182-5p and miR-23a-3p *in vitro* and *in vivo* resulted in a reversal of the progression of lipopolysaccharide-induced lung injury and fibrosis in MLE-12 cells and mice (114).

### 4.6 MiR-199a-5p

It has been shown that miR-199a-5p is significantly increased in the serum of IPF patients compared to healthy controls (115). MiR-199a-5p regulates autophagy and mediates mesenchymal stem cell (MSC) senescence in IPF patients by targeting the Sirt1/AMPK signaling pathway, inhibition of miR-199a-5p rejuvenated IPF-MSCs and increased their capacity to prevent lung fibrosis progression induced by bleomycin in mice (116). Lung fibroblasts overexpressing miR-199a-5p can increase SMAD4 expression, and miR-199a-5p can directly inhibit Caveolin-1 (CAV1 is a key effector of TGF-*β* signaling in lung fibroblasts) in lung fibroblasts, which can stimulate their proliferation, migration, invasion, and differentiation into myofibroblasts, thus promoting fibrotic (117).

## 5 Conclusion and perspective

TGF-*β* is a crucial instigator of pulmonary fibrogenesis and a principal mediator in the progression of PF. The activation of the TGF-β signaling pathway can catalyze the overexpression of profibrotic genes within the organism from various angles, culminating in fibrotic alterations within lung tissues. MiRNAs hold a pivotal role in the progression of PF. The existing findings underline the dual effects of miRNAs, suggesting that they can be utilized not only as diagnostic markers for PF but also as therapeutic targets for the treatment of diseases associated with PF. Of note, therapeutic miRNAs have been investigated in clinical trial stages (118). In the context of escalating disease severity, there are dramatic changes in the gene expression regulation patterns based on down-regulated miRNAs. The miR-29 family plays a dominant role in the early stages of PF, whereas the let-7 family takes precedence in the later stages of this disease (66).

In this comprehensive review, the intricate interplay between the TGF-*β* signaling mechanism and miRNAs in the progression of PF is elucidated. Antifibrotic miRNAs, such as miR-133a, miR-29, miR-130a-3p, miR-200, miR-9-5p and miR-26a, can inhibit the TGF-*β* signaling pathway and deter the progression of PF by acting on the positive regulators of signal transduction. Conversely, profibrotic miRNAs, such as miR-21, mir-145, miR-155, miR-424, miR-182-5p and miR-199a-5p, can enhance the TGF-*β* signaling pathway by repressing relevant negative regulators, thereby promoting the PF phenotype. Interestingly, there are also complex feedback regulatory loops. MiR-133a, miR-29, and miR-9-5p form a negative feedback loop with TGF-*β*-linked molecules, thus ameliorating PF. Simultaneously, miR-21, mir-145, miR-155, miR-424, and miR-182-5p generate a positive feedback loop with TGF-β-associated molecules, which promotes the progression of PF.

Many of the concerns about drugs with miRNAs are based on the fact that miRNA targeting has a broader and less specific mechanism that can easily lead to off-target effects compared to siRNA targeting, which has a high specificity. This factor is mainly due to incomplete binding at the 3’-UTR of the target sequence, especially at the seed region (119). Some miRNAs (within the same family) share a common seed region (bases 2–7 at the 5′ end), which is a key region for mRNA target recognition (120), and thus miRNA family members can target and repress the same genes. MiRNA therapeutics do not necessarily confine their effects to the intended tissues or cells, but they may also cause systemic side effects. For example, MRX34, a synthetic miR-34a mimic. MiR-34a not only acts as a tumor suppressor, but also affects immune cell signaling. MiR-34a is not only uptaken in tumor tissues, but also similarly uptaken in the bone marrow and the spleen, producing severe immune-associated side effects (121, 122).

Potential off-target effects and immunostimulation of miRNA-based drugs should be rigorously and extensively studied before application to animal models or human use. Overall, further unraveling the dynamic interaction between miRNAs via the TGF-*β* signaling pathway remains an indispensable trajectory for the development of novel antifibrotic treatment modalities.

## References

[1] Koudstaal T Funke-Chambour M Kreuter M Molyneaux PL Wijsenbeek MS. Pulmonary fibrosis: from pathogenesis to clinical decision-making. Trends Mol Med. (2023) 29:1076–87. doi: 10.1016/j.molmed.2023.08.010, PMID: 37716906

[2] Richeldi L Collard HR Jones MG. Idiopathic pulmonary fibrosis. Lancet. (2017) 389:1941–52. doi: 10.1016/S0140-6736(17)30866-8, PMID: 28365056

[3] Maher TM Bendstrup E Dron L Langley J Smith G Khalid JM. Global incidence and prevalence of idiopathic pulmonary fibrosis. Respir Res. (2021) 22:197. doi: 10.1186/s12931-021-01791-z, PMID: 34233665 PMC8261998

[4] Raghu G Remy-Jardin M Richeldi L Thomson CC Inoue Y Johkoh T. Idiopathic pulmonary fibrosis (an update) and progressive pulmonary fibrosis in adults: an official ATS/ERS/JRS/ALAT clinical practice guideline. Am J Respir Crit Care Med. (2022) 205:e18–47. doi: 10.1164/rccm.202202-0399ST, PMID: 35486072 PMC9851481

[5] Vancheri C Kreuter M Richeldi L Ryerson CJ Valeyre D Grutters JC. Nintedanib with add-on Pirfenidone in idiopathic pulmonary fibrosis. Results of the INJOURNEY trial. Am J Respir Crit Care Med. (2018) 197:356–63. doi: 10.1164/rccm.201706-1301OC, PMID: 28889759

[6] Galli JA Pandya A Vega-Olivo M Dass C Zhao H Criner GJ. Pirfenidone and nintedanib for pulmonary fibrosis in clinical practice: tolerability and adverse drug reactions. Respirology (Carlton, Vic). (2017) 22:1171–8. doi: 10.1111/resp.13024, PMID: 28317233

[7] Borie R Justet A Beltramo G Manali ED Pradère P Spagnolo P. Pharmacological management of IPF. Respirology (Carlton, Vic). (2016) 21:615–25. doi: 10.1111/resp.12778, PMID: 27072575

[8] Rock JR Barkauskas CE Cronce MJ Xue Y Harris JR Liang J. Multiple stromal populations contribute to pulmonary fibrosis without evidence for epithelial to mesenchymal transition. Proc Natl Acad Sci U S A. (2011) 108:E1475–83. doi: 10.1073/pnas.1117988108, PMID: 22123957 PMC3248478

[9] Frangogiannis N. Transforming growth factor-β in tissue fibrosis. J Exp Med. (2020) 217:e20190103. doi: 10.1084/jem.20190103, PMID: 32997468 PMC7062524

[10] Meng XM Nikolic-Paterson DJ Lan HY. TGF-β: the master regulator of fibrosis. Nat Rev Nephrol. (2016) 12:325–38. doi: 10.1038/nrneph.2016.48, PMID: 27108839

[11] Heldin CH Moustakas A. Signaling receptors for TGF-β family members. Cold spring Harb Perspect Biol. (2016) 8:a022053. doi: 10.1101/cshperspect.a022053 27481709 PMC4968163

[12] Vander Ark A Cao J Li X. TGF-β receptors: in and beyond TGF-β signaling. Cell Signal. (2018) 52:112–20. doi: 10.1016/j.cellsig.2018.09.002, PMID: 30184463

[13] Peng D Fu M Wang M Wei Y Wei X. Targeting TGF-β signal transduction for fibrosis and cancer therapy. Mol Cancer. (2022) 21:104. doi: 10.1186/s12943-022-01569-x, PMID: 35461253 PMC9033932

[14] Xiao L Du Y Shen Y He Y Zhao H Li Z. TGF-beta 1 induced fibroblast proliferation is mediated by the FGF-2/ERK pathway. Front Biosci. (2012) 17:2667–74. doi: 10.2741/4077 PMID: 22652804

[15] Sun X Xie Z Ma Y Pan X Wang J Chen Z. TGF-β inhibits osteogenesis by upregulating the expression of ubiquitin ligase SMURF1 via MAPK-ERK signaling. J Cell Physiol. (2018) 233:596–606. doi: 10.1002/jcp.25920, PMID: 28322449

[16] Hashimoto S Gon Y Takeshita I Matsumoto K Maruoka S Horie T. Transforming growth factor-beta1 induces phenotypic modulation of human lung fibroblasts to myofibroblast through a c-Jun-NH2-terminal kinase-dependent pathway. Am J Respir Crit Care Med. (2001) 163:152–7. doi: 10.1164/ajrccm.163.1.2005069 PMID: 11208641

[17] Pardoux C Derynck R. JNK regulates expression and autocrine signaling of TGF-beta1. Mol Cell. (2004) 15:170–1. doi: 10.1016/j.molcel.2004.07.001 PMID: 15260968

[18] Yu L Hébert MC Zhang YE. TGF-beta receptor-activated p38 MAP kinase mediates Smad-independent TGF-beta responses. EMBO J. (2002) 21:3749–59. doi: 10.1093/emboj/cdf366 12110587 PMC126112

[19] Wang Y Mack JA Hascall VC Maytin EV. Transforming growth factor-β receptor-mediated, p38 mitogen-activated protein kinase-dependent signaling drives enhanced Myofibroblast differentiation during skin wound healing in mice lacking Hyaluronan synthases 1 and 3. Am J Pathol. (2022) 192:1683–98. doi: 10.1016/j.ajpath.2022.08.003, PMID: 36063901 PMC9765314

[20] Ungefroren H Witte D Lehnert H. The role of small GTPases of the rho/Rac family in TGF-β-induced EMT and cell motility in cancer. Dev Dyn. (2018) 247:451–61. doi: 10.1002/dvdy.24505 28390160

[21] Melzer C Hass R Von Der Ohe J Lehnert H Ungefroren H. The role of TGF-β and its crosstalk with RAC1/RAC1b signaling in breast and pancreas carcinoma. Cell Commun Signal. (2017) 15:19. doi: 10.1186/s12964-017-0175-0, PMID: 28499439 PMC5429551

[22] Hao X Jin Y Zhang Y Li S Cui J He H. Inhibition of oncogenic Src ameliorates silica-induced pulmonary fibrosis via PI3K/AKT pathway. Int J Mol Sci. (2023) 24:774. doi: 10.3390/ijms24010774 36614217 PMC9821169

[23] Xiao Q Xiao J Liu J Liu J Shu G Yin G. Metformin suppresses the growth of colorectal cancer by targeting INHBA to inhibit TGF-β/PI3K/AKT signaling transduction. Cell Death Dis. (2022) 13:202. doi: 10.1038/s41419-022-04649-4, PMID: 35236827 PMC8891354

[24] Chanda D Otoupalova E Smith SR Volckaert T de Langhe SP Thannickal VJ. Developmental pathways in the pathogenesis of lung fibrosis. Mol Asp Med. (2019) 65:56–69. doi: 10.1016/j.mam.2018.08.004, PMID: 30130563 PMC6374163

[25] Libra A Sciacca E Muscato G Sambataro G Spicuzza L Vancheri C. Highlights on future treatments of IPF: clues and pitfalls. Int J Mol Sci. (2024) 25:8392. doi: 10.3390/ijms25158392 39125962 PMC11313529

[26] Bergeron A Soler P Kambouchner M Loiseau P Milleron B Valeyre D. Cytokine profiles in idiopathic pulmonary fibrosis suggest an important role for TGF-beta and IL-10. Eur Respir J. (2003) 22:69–76. doi: 10.1183/09031936.03.00014703 PMID: 12882453

[27] Gauldie J Bonniaud P Sime P Ask K Kolb M. TGF-beta, Smad3 and the process of progressive fibrosis. Biochem Soc Trans. (2007) 35:661–4. doi: 10.1042/BST0350661 PMID: 17635115

[28] Kim J Jeon S Kang SJ Kim KR Thai HBD Lee S. Lung-targeted delivery of TGF-β antisense oligonucleotides to treat pulmonary fibrosis. J Control Release. (2020) 322:108–21. doi: 10.1016/j.jconrel.2020.03.016, PMID: 32179111

[29] Krol J Loedige I Filipowicz W. The widespread regulation of microRNA biogenesis, function and decay. Nat Rev Genet. (2010) 11:597–610. doi: 10.1038/nrg2843, PMID: 20661255

[30] Kim VN. MicroRNA biogenesis: coordinated cropping and dicing. Nat Rev Mol Cell Biol. (2005) 6:376–85. doi: 10.1038/nrm1644 PMID: 15852042

[31] Seitz H Zamore PD. Rethinking the microprocessor. Cell. (2006) 125:827–9. doi: 10.1016/j.cell.2006.05.018 PMID: 16751089

[32] Khvorova A Reynolds A Jayasena SD. Functional siRNAs and miRNAs exhibit strand bias. Cell. (2003) 115:209–16. doi: 10.1016/s0092-8674(03)00801-8. PMID: 14567918

[33] Wu PH Zamore PD. To degrade a MicroRNA, destroy its Argonaute protein. Mol Cell. (2021) 81:223–5. doi: 10.1016/j.molcel.2020.12.043, PMID: 33482091

[34] Golden RJ Chen B Li T Braun J Manjunath H Chen X. An Argonaute phosphorylation cycle promotes microRNA-mediated silencing. Nature. (2017) 542:197–202. doi: 10.1038/nature21025, PMID: 28114302 PMC5302127

[35] Zhang J Li S Li L Li M Guo C Yao J. Exosome and exosomal microRNA: trafficking, sorting, and function. Genomics Proteomics Bioinformatics. (2015) 13:17–24. doi: 10.1016/j.gpb.2015.02.001, PMID: 25724326 PMC4411500

[36] Vickers KC Palmisano BT Shoucri BM Shamburek RD Remaley AT. MicroRNAs are transported in plasma and delivered to recipient cells by high-density lipoproteins. Nat Cell Biol. (2011) 13:423–33. doi: 10.1038/ncb2210, PMID: 21423178 PMC3074610

[37] Tabet F Vickers KC Cuesta Torres LF Wiese CB Shoucri BM Lambert G. HDL-transferred microRNA-223 regulates ICAM-1 expression in endothelial cells. Nat Commun. (2014) 5:3292. doi: 10.1038/ncomms4292 24576947 PMC4189962

[38] Arroyo JD Chevillet JR Kroh EM Ruf IK Pritchard CC Gibson DF. Argonaute2 complexes carry a population of circulating microRNAs independent of vesicles in human plasma. Proc Natl Acad Sci U S A. (2011) 108:5003–8. doi: 10.1073/pnas.1019055108, PMID: 21383194 PMC3064324

[39] Michell DL Vickers KC. HDL and microRNA therapeutics in cardiovascular disease. Pharmacol Ther. (2016) 168:43–52. doi: 10.1016/j.pharmthera.2016.09.001, PMID: 27595929 PMC5140688

[40] Wonnacott A Denby L Coward RJM Fraser DJ Bowen T. MicroRNAs and their delivery in diabetic fibrosis. Adv Drug Deliv Rev. (2022) 182:114045. doi: 10.1016/j.addr.2021.114045 34767865

[41] Liu M Cho WC Flynn RJ Jin X Song H Zheng Y. microRNAs in parasite-induced liver fibrosis: from mechanisms to diagnostics and therapeutics. Trends Parasitol. (2023) 39:859–72. doi: 10.1016/j.pt.2023.07.001, PMID: 37516634

[42] Negrete-García MC De Jesús Ramos-Abundis J Alvarado-Vasquez N Montes-Martínez E Montaño M Ramos C. Exosomal Micro-RNAs as intercellular communicators in idiopathic pulmonary fibrosis. Int J Mol Sci. (2022) 23:11047. doi: 10.3390/ijms231911047 36232350 PMC9569972

[43] Zhao Y Du D Chen S Chen Z Zhao J. New insights into the functions of MicroRNAs in cardiac fibrosis: from mechanisms to therapeutic strategies. Genes. (2022) 13:1390. doi: 10.3390/genes13081390 36011301 PMC9407613

[44] Lu TX Rothenberg ME. MicroRNA. J Allergy Clin Immunol. (2018) 141:1202–7. doi: 10.1016/j.jaci.2017.08.034, PMID: 29074454 PMC5889965

[45] Kadota T Fujita Y Araya J Watanabe N Fujimoto S Kawamoto H. Human bronchial epithelial cell-derived extracellular vesicle therapy for pulmonary fibrosis via inhibition of TGF-β-WNT crosstalk. J Extracell Vesicles. (2021) 10:e12124. doi: 10.1002/jev2.12124, PMID: 34377373 PMC8329991

[46] Chou MY Hsieh PL Chao SC Liao YW Yu CC Tsai CY. MiR-424/TGIF2-mediated pro-Fibrogenic responses in Oral submucous fibrosis. Int J Mol Sci. (2023) 24:5811. doi: 10.3390/ijms24065811 36982885 PMC10053232

[47] Feng Y Bao Y Ding J Li H Liu W Wang X. MicroRNA-130a attenuates cardiac fibrosis after myocardial infarction through TGF-β/Smad signaling by directly targeting TGF-β receptor 1. Bioengineered. (2022) 13:5779–91. doi: 10.1080/21655979.2022.2033380, PMID: 35188441 PMC8973730

[48] Das S Kumar M Negi V Pattnaik B Prakash YS Agrawal A. MicroRNA-326 regulates profibrotic functions of transforming growth factor-β in pulmonary fibrosis. Am J Respir Cell Mol Biol. (2014) 50:882–92. doi: 10.1165/rcmb.2013-0195OC, PMID: 24279830 PMC4068942

[49] Davis BN Hilyard AC Lagna G Hata A. SMAD proteins control DROSHA-mediated microRNA maturation. Nature. (2008) 454:56–61. doi: 10.1038/nature07086, PMID: 18548003 PMC2653422

[50] Marquez RT Bandyopadhyay S Wendlandt EB Keck K Hoffer BA Icardi MS. Correlation between microRNA expression levels and clinical parameters associated with chronic hepatitis C viral infection in humans. Lab Investig. (2010) 90:1727–36. doi: 10.1038/labinvest.2010.126, PMID: 20625373

[51] Ottaviani S Stebbing J Frampton AE Zagorac S Krell J de Giorgio A. TGF-β induces miR-100 and miR-125b but blocks let-7a through LIN28B controlling PDAC progression. Nat Commun. (2018) 9:1845. doi: 10.1038/s41467-018-03962-x, PMID: 29748571 PMC5945639

[52] Yang L Du X Liu L Cao Q Pan Z Li Q. miR-1306 mediates the feedback regulation of the TGF-β/SMAD signaling pathway in granulosa cells. Cells. (2019) 8:298. doi: 10.3390/cells8040298 30935128 PMC6523565

[53] Li N Zhou H Tang Q. miR-133: a suppressor of cardiac remodeling? Front Pharmacol. (2018) 9:903. doi: 10.3389/fphar.2018.00903, PMID: 30174600 PMC6107689

[54] Roderburg C Luedde M Vargas Cardenas D Vucur M Mollnow T Zimmermann HW. miR-133a mediates TGF-β-dependent derepression of collagen synthesis in hepatic stellate cells during liver fibrosis. J Hepatol. (2013) 58:736–42. doi: 10.1016/j.jhep.2012.11.022, PMID: 23183523

[55] Wei P Xie Y Abel PW Huang Y Ma Q Li L. Transforming growth factor (TGF)-β1-induced miR-133a inhibits myofibroblast differentiation and pulmonary fibrosis. Cell Death Dis. (2019) 10:670. doi: 10.1038/s41419-019-1873-x, PMID: 31511493 PMC6739313

[56] Jin ZQ. MicroRNA targets and biomarker validation for diabetes-associated cardiac fibrosis. Pharmacol Res. (2021) 174:105941. doi: 10.1016/j.phrs.2021.105941, PMID: 34656765

[57] Huang Y Wang Y Lin L Wang P Jiang L Liu J. Overexpression of miR-133a-3p inhibits fibrosis and proliferation of keloid fibroblasts by regulating IRF5 to inhibit the TGF-β/Smad2 pathway. Mol Cell Probes. (2020) 52:101563. doi: 10.1016/j.mcp.2020.101563, PMID: 32205184

[58] Bhandari V Hoey C Liu LY Lalonde E Ray J Livingstone J. Molecular landmarks of tumor hypoxia across cancer types. Nat Genet. (2019) 51:308–18. doi: 10.1038/s41588-018-0318-2, PMID: 30643250

[59] Wang G Wang F Zhang L Yan C Zhang Y. miR-133a silencing rescues glucocorticoid-induced bone loss by regulating the MAPK/ERK signaling pathway. Stem Cell Res Ther. (2021) 12:215. doi: 10.1186/s13287-021-02278-w, PMID: 33781345 PMC8008567

[60] Li Q Li M Zheng K Li H Yang H Ma S. Detection of microRNA expression levels based on microarray analysis for classification of idiopathic pulmonary fibrosis. Exp Ther Med. (2020) 20:3096–103. doi: 10.3892/etm.2020.9068, PMID: 32855677 PMC7444334

[61] Wang M Huo Z He X Liu F Liang J Wu L. The role of MiR-29 in the mechanism of fibrosis. Mini Rev Med Chem. (2023) 23:1846–58. doi: 10.2174/1389557523666230328125031, PMID: 37018517

[62] Cushing L Kuang PP Qian J Shao F Wu J Little F. miR-29 is a major regulator of genes associated with pulmonary fibrosis. Am J Respir Cell Mol Biol. (2011) 45:287–94. doi: 10.1165/rcmb.2010-0323OC, PMID: 20971881 PMC3175558

[63] Xiao J Meng XM Huang XR Chung AC Feng YL Hui DS. miR-29 inhibits bleomycin-induced pulmonary fibrosis in mice. Mol Ther. (2012) 20:1251–60. doi: 10.1038/mt.2012.36, PMID: 22395530 PMC3369297

[64] Chioccioli M Roy S Newell R Pestano L Dickinson B Rigby K. A lung targeted miR-29 mimic as a therapy for pulmonary fibrosis. EBioMedicine. (2022) 85:104304. doi: 10.1016/j.ebiom.2022.104304, PMID: 36265417 PMC9587275

[65] Mai C Verleden SE Mcdonough JE Willems S De Wever W Coolen J. Thin-section CT features of idiopathic pulmonary fibrosis correlated with Micro-CT and histologic analysis. Radiology. (2017) 283:252–63. doi: 10.1148/radiol.2016152362, PMID: 27715655 PMC5375628

[66] McDonough JE Ahangari F Li Q Jain S Verleden SE Herazo-Maya J. Transcriptional regulatory model of fibrosis progression in the human lung. JCI. Insight. (2019) 4. doi: 10.1172/jci.insight.131597 PMC6948862 31600171

[67] Liu L Wang P Wang YS Zhang YN Li C Yang ZY. MiR-130a-3p alleviates liver fibrosis by suppressing HSCs activation and skewing macrophage to Ly6C(lo) phenotype. Front Immunol. (2021) 12:696069. doi: 10.3389/fimmu.2021.696069, PMID: 34421906 PMC8375151

[68] Ai K Zhu X Kang Y Li H Zhang L. miR-130a-3p inhibition protects against renal fibrosis in vitro via the TGF-β1/Smad pathway by targeting SnoN. Exp Mol Pathol. (2020) 112:104358. doi: 10.1016/j.yexmp.2019.104358 31836508

[69] Su S Zhao Q He C Huang D Liu J Chen F. miR-142-5p and miR-130a-3p are regulated by IL-4 and IL-13 and control profibrogenic macrophage program. Nat Commun. (2015) 6:8523. doi: 10.1038/ncomms9523, PMID: 26436920 PMC4600756

[70] Shi J Chen M Ouyang L Wang Q Guo Y Huang L. miR-142-5p and miR-130a-3p regulate pulmonary macrophage polarization and asthma airway remodeling. Immunol Cell Biol. (2020) 98:715–25. doi: 10.1111/imcb.12369, PMID: 32524675

[71] Liu Y Ding Y Hou Y Yu T Nie H Cui Y. The miR-130a-3p/TGF-βRII Axis participates in inhibiting the differentiation of fibroblasts induced by TGF-β1. Front Pharmacol. (2021) 12:732540. doi: 10.3389/fphar.2021.732540, PMID: 34393805 PMC8355625

[72] Ding Y Hou Y Liu Y Yu T Cui Y Nie H. MiR-130a-3p alleviates inflammatory and fibrotic phases of pulmonary fibrosis through Proinflammatory factor TNF-α and Profibrogenic receptor TGF-βRII. Front Pharmacol. (2022) 13:863646. doi: 10.3389/fphar.2022.863646, PMID: 35431964 PMC9006815

[73] Yang S Banerjee S de Freitas A Sanders YY Ding Q Matalon S. Participation of miR-200 in pulmonary fibrosis. Am J Pathol. (2012) 180:484–93. doi: 10.1016/j.ajpath.2011.10.005, PMID: 22189082 PMC3349843

[74] Mansour SM El-Abhar HS Soubh AA. MiR-200a inversely correlates with hedgehog and TGF-β canonical/non-canonical trajectories to orchestrate the anti-fibrotic effect of Tadalafil in a bleomycin-induced pulmonary fibrosis model. Inflammopharmacology. (2021) 29:167–82. doi: 10.1007/s10787-020-00748-w, PMID: 32914382

[75] Chilosi M Caliò A Rossi A Gilioli E Pedica F Montagna L. Epithelial to mesenchymal transition-related proteins ZEB1, β-catenin, and β-tubulin-III in idiopathic pulmonary fibrosis. Mod Pathol. (2017) 30:26–38. doi: 10.1038/modpathol.2016.147, PMID: 27586205

[76] Fierro-Fernández M Miguel V Márquez-Expósito L Nuevo-Tapioles C Herrero JI Blanco-Ruiz E. MiR-9-5p protects from kidney fibrosis by metabolic reprogramming. FASEB J. (2020) 34:410–31. doi: 10.1096/fj.201901599RR, PMID: 31914684

[77] Zhou Z Zhang R Li X Zhang W Zhan Y Lang Z. Circular RNA cVIM promotes hepatic stellate cell activation in liver fibrosis via miR-122-5p/miR-9-5p-mediated TGF-β signaling cascade. Commun Biol. (2024) 7:113. doi: 10.1038/s42003-024-05797-3, PMID: 38243118 PMC10798957

[78] Xiao Y Zhang Y Chen Y Li J Zhang Z Sun Y. Inhibition of MicroRNA-9-5p protects against cardiac remodeling following myocardial infarction in mice. Hum Gene Ther. (2019) 30:286–301. doi: 10.1089/hum.2018.059, PMID: 30101604

[79] Zhang Y Yao XH Wu Y Cao GK Han D. LncRNA NEAT1 regulates pulmonary fibrosis through miR-9-5p and TGF-β signaling pathway. Eur Rev Med Pharmacol Sci. (2020) 24:8483–92. doi: 10.26355/eurrev_202008_22661, PMID: 32894569

[80] Fierro-Fernández M Busnadiego Ó Sandoval P Espinosa-Díez C Blanco-Ruiz E Rodríguez M. miR-9-5p suppresses pro-fibrogenic transformation of fibroblasts and prevents organ fibrosis by targeting NOX4 and TGFBR2. EMBO Rep. (2015) 16:1358–77. doi: 10.15252/embr.201540750, PMID: 26315535 PMC4766462

[81] Zheng H Ji J Zhao T Wang E Zhang A. Exosome-encapsulated miR-26a attenuates aldosterone-induced tubulointerstitial fibrosis by inhibiting the CTGF/SMAD3 signaling pathway. Int J Mol Med. (2023) 51:11. doi: 10.3892/ijmm.2022.5214 36524378 PMC9848436

[82] Chen X Xiao W Chen W Liu X Wu M Bo Q. MicroRNA-26a and -26b inhibit lens fibrosis and cataract by negatively regulating Jagged-1/notch signaling pathway. Cell Death Differ. (2017) 24:1431–42. doi: 10.1038/cdd.2016.152, PMID: 28622289 PMC5520447

[83] Liang H Liu S Chen Y Bai X Liu L Dong Y. miR-26a suppresses EMT by disrupting the Lin28B/let-7d axis: potential cross-talks among miRNAs in IPF. J Mol Med (Berl). (2016) 94:655–65. doi: 10.1007/s00109-016-1381-8, PMID: 26787543

[84] Liang H Xu C Pan Z Zhang Y Xu Z Chen Y. The antifibrotic effects and mechanisms of microRNA-26a action in idiopathic pulmonary fibrosis. Mol Ther. (2014) 22:1122–33. doi: 10.1038/mt.2014.42, PMID: 24594795 PMC4048895

[85] Climent M Viggiani G Chen YW Coulis G Castaldi A. MicroRNA and ROS crosstalk in cardiac and pulmonary diseases. Int J Mol Sci. (2020) 21. doi: 10.3390/ijms21124370, PMID: 32575472 PMC7352701

[86] Specjalski K Niedoszytko M. MicroRNAs: future biomarkers and targets of therapy in asthma? Curr Opin Pulm Med. (2020) 26:285–92. doi: 10.1097/MCP.0000000000000673, PMID: 32101904

[87] Lu Y Liu Z Zhang Y Wu X Bian W Shan S. METTL3-mediated m6A RNA methylation induces the differentiation of lung resident mesenchymal stem cells into myofibroblasts via the miR-21/PTEN pathway. Respir Res. (2023) 24:300. doi: 10.1186/s12931-023-02606-z, PMID: 38017523 PMC10683095

[88] Bica-Pop C Cojocneanu-Petric R Magdo L Raduly L Gulei D Berindan-Neagoe I. Overview upon miR-21 in lung cancer: focus on NSCLC. Cell Mol Life Sci. (2018) 75:3539–51. doi: 10.1007/s00018-018-2877-x, PMID: 30030592 PMC11105782

[89] Liu G Friggeri A Yang Y Milosevic J Ding Q Thannickal VJ. miR-21 mediates fibrogenic activation of pulmonary fibroblasts and lung fibrosis. J Exp Med. (2010) 207:1589–97. doi: 10.1084/jem.20100035, PMID: 20643828 PMC2916139

[90] Liu L Yin H Huang M He J Yi G Wang Z. miR-21 promotes pulmonary fibrosis in rats via down-regulating the expression of ADAMTS-1. Xi Bao Yu Fen Zi Mian Yi Xue Za Zhi. (2016) 32:1636–40. PMID: 27916096

[91] Zhou J Xu Q Zhang Q Wang Z Guan S. A novel molecular mechanism of microRNA-21 inducing pulmonary fibrosis and human pulmonary fibroblast extracellular matrix through transforming growth factor β1-mediated SMADs activation. J Cell Biochem. (2018) 119:7834–43. doi: 10.1002/jcb.27185, PMID: 29943845

[92] Sato S Chong SG Upagupta C Yanagihara T Saito T Shimbori C. Fibrotic extracellular matrix induces release of extracellular vesicles with pro-fibrotic miRNA from fibrocytes. Thorax. (2021) 76:895–906. doi: 10.1136/thoraxjnl-2020-215962, PMID: 33859055

[93] Konkol M Śniatała P Milecki P. Radiation-induced lung injury - what do we know in the era of modern radiotherapy? Rep Pract Oncol Radiother. (2022) 27:552–65. doi: 10.5603/RPOR.a2022.0046, PMID: 36186693 PMC9518776

[94] Yi M Yuan Y Ma L Li L Qin W Wu B. Inhibition of TGFβ1 activation prevents radiation-induced lung fibrosis. Clin Transl Med. (2024) 14:e1546. doi: 10.1002/ctm2.1546, PMID: 38239077 PMC10797247

[95] Kwon OS Kim KT Lee E Kim M Choi SH Li H. Induction of MiR-21 by stereotactic body radiotherapy contributes to the pulmonary fibrotic response. PLoS One. (2016) 11:e0154942. doi: 10.1371/journal.pone.0154942, PMID: 27171163 PMC4865046

[96] Kent OA McCall MN Cornish TC Halushka MK. Lessons from miR-143/145: the importance of cell-type localization of miRNAs. Nucleic Acids Res. (2014) 42:7528–38. doi: 10.1093/nar/gku461, PMID: 24875473 PMC4081080

[97] Rangrez AY Massy ZA Metzinger-le Meuth V Metzinger L. miR-143 and miR-145: molecular keys to switch the phenotype of vascular smooth muscle cells. Circ Cardiovasc Genet. (2011) 4:197–205. doi: 10.1161/CIRCGENETICS.110.958702, PMID: 21505201

[98] Cordes KR Sheehy NT White MP Berry EC Morton SU Muth AN. miR-145 and miR-143 regulate smooth muscle cell fate and plasticity. Nature. (2009) 460:705–10. doi: 10.1038/nature08195, PMID: 19578358 PMC2769203

[99] Guiot J Henket M Remacle C Cambier M Struman I Winandy M. Systematic review of overlapping microRNA patterns in COVID-19 and idiopathic pulmonary fibrosis. Respir Res. (2023) 24:112. doi: 10.1186/s12931-023-02413-6, PMID: 37061683 PMC10105547

[100] Yang S Cui H Xie N Icyuz M Banerjee S Antony VB. miR-145 regulates myofibroblast differentiation and lung fibrosis. FASEB J. (2013) 27:2382–91. doi: 10.1096/fj.12-219493, PMID: 23457217 PMC3659354

[101] Bala S Csak T Saha B Zatsiorsky J Kodys K Catalano D. The pro-inflammatory effects of miR-155 promote liver fibrosis and alcohol-induced steatohepatitis. J Hepatol. (2016) 64:1378–87. doi: 10.1016/j.jhep.2016.01.035, PMID: 26867493 PMC4874886

[102] Eissa MG Artlett CM. The MicroRNA miR-155 is essential in fibrosis. Noncoding. RNA. (2019) 5:23. doi: 10.3390/ncrna5010023 PMC6468348 30871125

[103] Kong W Yang H He L Zhao JJ Coppola D Dalton WS. MicroRNA-155 is regulated by the transforming growth factor beta/Smad pathway and contributes to epithelial cell plasticity by targeting RhoA. Mol Cell Biol. (2008) 28:6773–84. doi: 10.1128/MCB.00941-08, PMID: 18794355 PMC2573297

[104] Sun X Kang Y Xue S Zou J Xu J Tang D. In vivo therapeutic success of MicroRNA-155 antagomir in a mouse model of pulmonary fibrosis induced by bleomycin. Korean J Intern Med. (2021) 36:S160–9. doi: 10.3904/kjim.2019.098, PMID: 32506869 PMC8009162

[105] Adel RM Helal H Ahmed Fouad M Sobhy Abd-Elhalem S. Regulation of miRNA-155-5p ameliorates NETosis in pulmonary fibrosis rat model via inhibiting its target cytokines IL-1β, TNF-α and TGF-β1. Int Immunopharmacol. (2024) 127:111456. doi: 10.1016/j.intimp.2023.111456, PMID: 38159555

[106] Dastmalchi N Baradaran B Banan Khojasteh SM Hosseinpourfeizi M Safaralizadeh R. miR-424: a novel potential therapeutic target and prognostic factor in malignancies. Cell Biol Int. (2021) 45:720–30. doi: 10.1002/cbin.11530, PMID: 33325141

[107] Ghafouri-Fard S Askari A Hussen BM Taheri M Akbari Dilmaghani N. Role of miR-424 in the carcinogenesis. Clin Transl Oncol. (2024) 26:16–38. doi: 10.1007/s12094-023-03209-2, PMID: 37178445 PMC10761534

[108] Xuan J Liu Y Zeng X Wang H. Sequence requirements for miR-424-5p regulating and function in cancers. Int J Mol Sci. (2022) 23. doi: 10.3390/ijms23074037, PMID: 35409396 PMC8999618

[109] Kadota T Yoshioka Y Fujita Y Araya J Minagawa S Hara H. Extracellular vesicles from fibroblasts induce epithelial-cell senescence in pulmonary fibrosis. Am J Respir Cell Mol Biol. (2020) 63:623–36. doi: 10.1165/rcmb.2020-0002OC, PMID: 32730709

[110] Huang Y Xie Y Abel PW Wei P Plowman J Toews ML. TGF-β1-induced miR-424 promotes pulmonary myofibroblast differentiation by targeting Slit2 protein expression. Biochem Pharmacol. (2020) 180:114172. doi: 10.1016/j.bcp.2020.114172, PMID: 32712053 PMC8742596

[111] Xiao X Huang C Zhao C Gou X Senavirathna LK Hinsdale M. Regulation of myofibroblast differentiation by miR-424 during epithelial-to-mesenchymal transition. Arch Biochem Biophys. (2015) 566:49–57. doi: 10.1016/j.abb.2014.12.007, PMID: 25524739 PMC4297572

[112] Chen Y Zhang Q Zhou Y Yang Z Tan M. Inhibition of miR-182-5p attenuates pulmonary fibrosis via TGF-β/Smad pathway. Hum Exp Toxicol. (2020) 39:683–95. doi: 10.1177/0960327119895549, PMID: 31884830

[113] Li JH Zhu HJ Huang XR Lai KN Johnson RJ Lan HY. Smad7 inhibits fibrotic effect of TGF-Beta on renal tubular epithelial cells by blocking Smad2 activation. J Am Soc Nephrol. (2002) 13:1464–72. doi: 10.1097/01.asn.0000014252.37680.e4 PMID: 12039975

[114] Xiao K He W Guan W Hou F Yan P Xu J. Mesenchymal stem cells reverse EMT process through blocking the activation of NF-κB and hedgehog pathways in LPS-induced acute lung injury. Cell Death Dis. (2020) 11:863. doi: 10.1038/s41419-020-03034-3, PMID: 33060560 PMC7567061

[115] Yang G Yang L Wang W Wang J Wang J Xu Z. Discovery and validation of extracellular/circulating microRNAs during idiopathic pulmonary fibrosis disease progression. Gene. (2015) 562:138–44. doi: 10.1016/j.gene.2015.02.065, PMID: 25725128

[116] Shi L Han Q Hong Y Li W Gong G Cui J. Inhibition of miR-199a-5p rejuvenates aged mesenchymal stem cells derived from patients with idiopathic pulmonary fibrosis and improves their therapeutic efficacy in experimental pulmonary fibrosis. Stem Cell Res Ther. (2021) 12:147. doi: 10.1186/s13287-021-02215-x 33632305 PMC7905557

[117] Lino Cardenas CL Henaoui IS Courcot E Roderburg C Cauffiez C Aubert S. miR-199a-5p is upregulated during fibrogenic response to tissue injury and mediates TGFbeta-induced lung fibroblast activation by targeting caveolin-1. PLoS Genet. (2013) 9:e1003291. doi: 10.1371/journal.pgen.1003291, PMID: 23459460 PMC3573122

[118] Hanna J Hossain GS Kocerha J. The potential for microRNA therapeutics and clinical research. Front Genet. (2019) 10:478. doi: 10.3389/fgene.2019.00478, PMID: 31156715 PMC6532434

[119] Segal M Slack FJ. Challenges identifying efficacious miRNA therapeutics for cancer. Expert Opin Drug Discov. (2020) 15:987–92. doi: 10.1080/17460441.2020.1765770, PMID: 32421364 PMC7415578

[120] Bartel DP. MicroRNAs: genomics, biogenesis, mechanism, and function. Cell. (2004) 116:281–97. doi: 10.1016/s0092-8674(04)00045-5 PMID: 14744438

[121] Hong DS Kang YK Borad M Sachdev J Ejadi S Lim HY. Phase 1 study of MRX34, a liposomal miR-34a mimic, in patients with advanced solid tumours. Br J Cancer. (2020) 122:1630–7. doi: 10.1038/s41416-020-0802-1, PMID: 32238921 PMC7251107

[122] Kelnar K Bader AG. A qRT-PCR method for determining the biodistribution profile of a miR-34a mimic. Methods Mol Biol. (2015) 1317:125–33. doi: 10.1007/978-1-4939-2727-2_8, PMID: 26072405

